# Efficacy and safety of neoadjuvant treatment with PD-1 inhibitor in locally advanced head and neck squamous cell carcinoma

**DOI:** 10.3389/fonc.2026.1887708

**Published:** 2026-08-26

**Authors:** Shaowei Xu, Hongxin Zheng, Xiaoqi Liao, Manyu Jiang, Hanwei Peng, Muyuan Liu

**Affiliations:** 1 Department of Head, Neck and Thyroid Surgery, Cancer Hospital of Shantou University Medical College, Shantou, China; 2 Department of Department of Otorhinolaryngology, The First Affiliated Hospital of Shantou University Medical College, Shantou, China; 3 Department of General Surgery, Puning People’s Hospital, Puning, China

**Keywords:** head and neck squamous cell carcinoma (HNSC), immunotherapy, neoadjuvant therapy (NACT), PD-1inhibitors, PD-L1 inhibitors

## Abstract

Head and neck squamous cell carcinoma (HNSCC) is a common malignant tumor. Traditional treatments have unstable efficacy, with risks of relapse, drug resistance and side effects. Immunotherapy has achieved good results in recurrent/metastatic advanced HNSCC, but relevant research on its neoadjuvant application is insufficient. This study retrospectively analyzes clinical data to evaluate the efficacy and safety of PD-1 inhibitors as neoadjuvant therapy for LA-HNSCC, providing a scientific basis for treatment strategies. This study conducted a retrospective analysis of patients with locally advanced head and neck squamous cell carcinoma (LA-HNSCC) treated at Shantou University Cancer Hospital from November 2020 to March 2024. The experimental group was administered neoadjuvant PD-1 inhibitor combined with chemotherapy, while the control group received chemotherapy alone. Efficacy was evaluated in accordance with RECIST 1.1 criteria, and treatment-related adverse events were graded based on CTCAE 5.0. SPSS 27.0 software and Kaplan-Meier method were employed for statistical analysis, with p<0.05 considered statistically significant. Baseline characteristics were well balanced between the two groups (P>0.05), with no significant difference in RECIST-based efficacy outcomes (P>0.05). The study group exhibited a significantly higher pathological downgrading rate and pathological complete response (pCR) (P = 0.032), a favorable safety profile, and improved overall survival (OS) (P = 0.044). Neoadjuvant immunochemotherapy was associated with improved overall survival, and surgery was associated with a lower risk of death. These findings are exploratory and require confirmation in larger prospective studies. Neoadjuvant immunochemotherapy was associated with improved pathological response and OS in LA-HNSCC, with an acceptable safety profile. These exploratory findings warrant confirmation in larger prospective studies.a retrospective analysis of patients with locally advanced head and neck squamous cell carcinoma (LA-HNSCC) treated at Shantou University Cancer Hospital from November 2020 to March 2024. The experimental group was administered neoadjuvant PD-1 inhibitor combined with chemotherapy, while the control group received chemotherapy alone. Efficacy was evaluated in accordance with RECIST 1.1 criteria, and treatment-related adverse events were graded based on CTCAE 5.0. SPSS 27.0 software and Kaplan-Meier method were employed for statistical analysis, with p<0.05 considered statistically significant. Baseline characteristics were well balanced between the two groups (P>0.05), with no significant difference in RECIST-based efficacy outcomes (P>0.05). The study group exhibited a significantly higher pathological downgrading rate and pathological complete response (pCR) (P = 0.032), a favorable safety profile, and improved overall survival (OS) (P = 0.044). Neoadjuvant treatment regimen and surgical intervention were identified as factors associated with improved OS. These should be interpreted as exploratory given the sample size and group imbalance. Neoadjuvant immunochemotherapy was associated with improved pathological response and OS in LA-HNSCC, with an acceptable safety profile. These exploratory findings warrant confirmation in larger prospective studies. A retrospective analysis of patients with locally advanced head and neck squamous cell carcinoma (LA-HNSCC) treated at Shantou University Cancer Hospital from November 2020 to March 2024. The experimental group was administered neoadjuvant PD-1 inhibitor combined with chemotherapy, while the control group received chemotherapy alone. Efficacy was evaluated in accordance with RECIST 1.1 criteria, and treatment-related adverse events were graded based on CTCAE 5.0. SPSS 27.0 software and Kaplan-Meier method were employed for statistical analysis, with p<0.05 considered statistically significant. Baseline characteristics were well balanced between the two groups (P>0.05), with no significant difference in RECIST-based efficacy outcomes (P>0.05). The study group exhibited a significantly higher pathological downgrading rate and pathological complete response (pCR) (P = 0.032), a favorable safety profile, and improved overall survival (OS) (P = 0.044). Neoadjuvant treatment regimen and surgical intervention were identified as factors associated with improved OS. These should be interpreted as exploratory given the sample size and group imbalance. Neoadjuvant immunochemotherapy was associated with improved pathological response and OS in LA-HNSCC, with an acceptable safety profile. These exploratory findings warrant confirmation in larger prospective studies. A retrospective analysis of patients with locally advanced head and neck squamous cell carcinoma (LA-HNSCC) treated at Shantou University Cancer Hospital from November 2020 to March 2024. The experimental group was administered neoadjuvant PD-1 inhibitor combined with chemotherapy, while the control group received chemotherapy alone. Efficacy was evaluated in accordance with RECIST 1.1 criteria, and treatment-related adverse events were graded based on CTCAE 5.0. SPSS 27.0 software and Kaplan-Meier method were employed for statistical analysis, with p<0.05 considered statistically significant. Baseline characteristics were well balanced between the two groups (P>0.05), with no significant difference in RECIST-based efficacy outcomes (P>0.05). The study group exhibited a significantly higher pathological downgrading rate and pathological complete response (pCR) (P = 0.032), a favorable safety profile, and improved overall survival (OS) (P = 0.044). Neoadjuvant immunochemotherapy was associated with improved overall survival, and surgery was associated with a lower risk of death. These findings are exploratory and require confirmation in larger prospective studies. Neoadjuvant immunochemotherapy was associated with improved pathological response and OS in LA-HNSCC, with an acceptable safety profile. These exploratory findings warrant confirmation in larger prospective studies.

## 1 Introduction

Head and neck squamous cell carcinoma (HNSCC) is the sixth most common cancer globally, originating from oral, pharyngeal and laryngeal mucosa, with rising incidence (1). Major risk factors include smoking, alcohol, betel nut use and HPV infection (2); HPV-positive cases are increasing while smoking-related cases decline (3). Anatomical complexity makes HNSCC severely impair speech, swallowing and quality of life. Nasopharyngeal carcinoma (NPC), highly prevalent in southern China (4), was excluded due to distinct radiotherapy-dominant management. Locally advanced HNSCC (LA-HNSCC) needs multidisciplinary therapy including surgery, radiotherapy, chemotherapy, targeted and immunotherapy.

Tumor immunotherapy activates anti-tumor immunity by reversing immune escape via checkpoint pathways (CTLA-4, PD-1/PD-L1) (5–9). PD-1/PD-L1 blockade key trials (KEYNOTE-040, 048) established anti-PD-1 superiority in recurrent/metastatic HNSCC (10, 11). Neoadjuvant immunotherapy for LA-HNSCC shows potential but remains understudied (12), especially in domestic real-world settings (13). Overseas neoadjuvant PD-1 inhibitor trials demonstrate promising pathological response and safety (14–18), but mechanisms, standardized regimens and real-world evidence are still insufficient (14). Despite global progress, key clinical issues remain unanswered. This study retrospectively evaluates neoadjuvant immunochemotherapy efficacy and safety in LA-HNSCC to support clinical application.

## 2 Material and methods

### 2.1 Clinical data

This retrospective study analyzed medical records of patients with LA-HNSCC treated at our institution from November 2020 to March 2024. Patients were divided into two groups according to the neoadjuvant regimen received: study group (PD-1 inhibitor plus platinum chemotherapy) and control group (platinum chemotherapy alone). Treatment allocation was determined at the discretion of the treating multidisciplinary team (MDT), comprising head and neck surgeons, medical oncologists, and radiation oncologists at Shantou University Medical College Cancer Hospital. The choice between neoadjuvant immunochemotherapy and chemotherapy alone was based on a combination of clinical factors, including tumor stage and resectability, patient performance status, comorbidities, and patient preference.

**Inclusion criteria**: 1) Age 18–75 years; 2) Histologically/pathologically confirmed locally advanced HNSCC; 3) Receipt of two neoadjuvant cycles with both post-cycle and pre-definitive-treatment imaging available; 4) ≥1 measurable lesion per RECIST 1.1 (assessed by CT or MRI).

**Exclusion criteria**: 1) Non-head and neck primary or non-squamous histology (including nasopharyngeal carcinoma); 2) Distant metastasis; 3) Incomplete clinical records.

### 2.2 Data collection

LA-HNSCC staging followed AJCC 8th edition (19, 20). Baseline data included gender, age, BMI, smoking and alcohol history. Clinical data comprised primary tumor site, pathological type, TNM stage, immune agent type, neoadjuvant cycle number and treatment regimen. Laboratory data included WBC, RBC, platelet count, creatinine, ALT, AST and thyroid function. Tumor and positive lymph node diameters on pre- and post-neoadjuvant CT/MRI were measured for efficacy. Surgical and perioperative pathological data were collected. Follow-up to March 1, 2025 included adverse reactions, recurrence/metastasis, quality of life and survival status via hospital records, outpatient review and telephone contact.

The neoadjuvant therapy regimens consisted of 1) the study group receiving PD-1 inhibitors combined with a platinum-based TP (Taxanes+ Platinum) chemotherapy regimen, and 2) the control group receiving platinum-based TP chemotherapy alone.

1. Study Group: ​ PD-1 inhibitor (Pembrolizumab, Penpulimab, or Tislelizumab) combined with platinum-based TP chemotherapy (Paclitaxel +Platinum). Each cycle lasted 3 weeks, and all medications were administered according to guidelines and drug package inserts (See **Table 1**).
2. Control Group: ​ Chemotherapy alone, specifically the platinum-based TP regimen.

**Table 1:** Neoadjuvant drugs.

Drug class	Drug	Dose
PD-1 inhibitor	Pembrolizumab	200mg
	Penpulimab	200mg
	Tislelizumab	200mg
Paclitaxel	Albumin-bound paclitaxel	260mg
Platinum-based	Cisplatin	75mg/m^2^
	Nedaplatin	80-100mg/m^2^

### 2.3 Efficacy and safety evaluation

#### 2.3.1 Efficacy evaluation

Tumor response was evaluated every two cycles using RECIST 1.1 (21), with at least one measurable target lesion required for neoadjuvant efficacy assessment. Responses were classified as CR, PR, SD or PD based on lesion changes.

Clinical-pathological downstaging was determined by comparing pre-treatment clinical TNM (AJCC 8th) with postoperative pathological TNM in surgical patients.

Pathological complete response (pCR) was defined as no residual viable tumor in resected specimens.

Patients lost to follow-up or without progression/death by study end were censored in survival analysis.

#### 2.3.2 Safety evaluation

Safety was evaluated by recording the type, frequency, and severity of adverse events (AEs) throughout the treatment period. AEs were graded according to the Common Terminology Criteria for Adverse Events (CTCAE) version 5.0 (22). Laboratory parameters, including hematological, hepatic, and renal function tests, as well as thyroid function, were monitored regularly. Any clinically significant abnormalities or AEs were documented and managed accordingly.

## 3 Statistical analysis

Data were analyzed using SPSS 27.0. Normally distributed measurement data were presented as mean ± standard deviation, and count data as frequency (%). Inter-group comparisons used t-test for measurement data and Chi-square or Fisher exact test for count data. Efficacy was assessed by RECIST 1.1, with waterfall plots for tumor regression. Survival analysis employed Kaplan-Meier curves for OS and PFS, compared by Log-rank test. All tests were two-tailed, with P < 0.05 considered statistically significant.

## 4 Results

### 4.1 Patient characteristics

**Figure 1:**
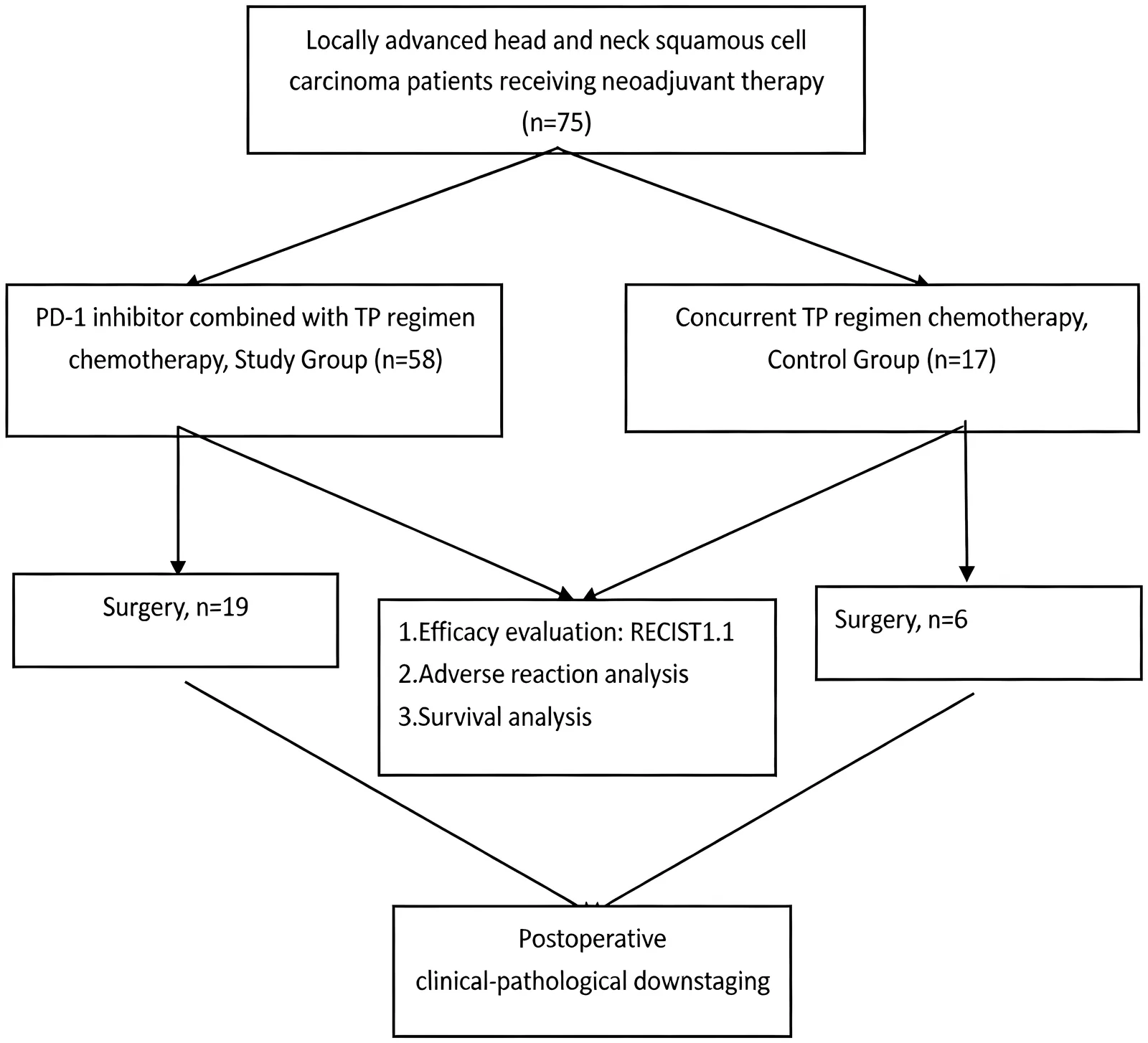
Research roadmap.

From the hospitalized cases between November 2020 and March 2024, a total of 75 patients who met the inclusion and exclusion criteria were enrolled. Among them, 58 patients (77.33%) were assigned to the study group, and 17 patients (22.67%) to the control group. The case grouping and assessment details are shown in **Figure 1**. Following neoadjuvant therapy, the subsequent definitive treatment modality (e.g., surgery or radical radiotherapy) was determined based on the degree of tumor downstaging. Patients who achieved a major partial response (major PR) or better after neoadjuvant therapy—defined as a reduction of more than 70% in the sum of the longest diameters of measurable lesions—were recommended for radical radiotherapy. Those who did not achieve a major PR were recommended for surgical resection.

In the study group (n=58), the median age was 61 years (range: 41–69 years), and the mean BMI was 20.56 ± 3.72 kg/m². The cohort comprised 52 males (89.66%) and 6 females (10.34%), resulting in a male-to-female ratio approximating 9:1. Thirty-seven patients (63.79%) had a history of smoking, and 28 (48.28%) had a history of alcohol consumption. Primary tumor sites included the oral cavity in 21 cases (66.67%), oropharynx in 17 (29.30%), hypopharynx in 14 (24.10%), and larynx in 6 (10.30%). Patients in the study group received neoadjuvant immunotherapy with PD-1 inhibitors, including tislelizumab (n=29, 50.0%), pembrolizumab (n=22, 37.9%), and penpulimab (n=7, 12.1%). Following neoadjuvant PD-1 inhibitor combined with chemotherapy, 22 patients (37.93%) underwent radical radiotherapy, 19 (33.33%) underwent surgical resection, and the remaining 17 patients, due to lack of downstaging, disease progression, or patient refusal of surgery/radiotherapy, received palliative treatment.

In the control group (n=17), the median age was 59 years (range: 31–75 years), with a mean BMI of 21.18 ± 2.79 kg/m² and a male-to-female ratio also approximating 9:1. Twelve patients (70.59%) had a smoking history, and 8 (47.06%) had an alcohol consumption history. Primary tumor sites were distributed as follows: oral cavity in 5 cases (29.40%), oropharynx in 7 (41.20%), hypopharynx in 4 (23.50%), and larynx in 1 (5.90%). After neoadjuvant chemotherapy, 5 patients (29.41%) underwent radical radiotherapy, 6 (35.29%) underwent surgery, and the remaining 6 patients, due to lack of downstaging, disease progression, or patient refusal of surgery/radiotherapy, received palliative treatment.

Baseline data were analyzed with SPSS 27.0. Comparisons showed no significant between-group differences in age, gender, BMI, smoking, alcohol use, radiotherapy or surgery rates (all P>0.05), confirming balanced baseline distribution. Patient clinical characteristics are summarized in **Table 2**.

**Table 2:** General data of 75 patients with LA HNSCC.

Item	Total (n=75)	Study group n=58	Control group n=17	t/χ²	P
Age (range,y)	61 (31~75)	61 (33~74)	59 (31~75)	χ²=0.488	0.626
BMI, Mean ± SD	20.51 ± 3.63	20.56 ± 3.72	20.34 ± 3.40	t=0.214	0.942
Sex, n (%) male female	67 (89.30) 8 (10.70)	52 (89.66) 6 (10.34)	15 (88.24) 2 (11.76)	-	1.000
Smoking, n (%) Y N	36 (48.00) 39 (52.00)	37 (63.79) 21 (36.21)	12 (70.59) 5 (29.41)	χ²=0.268	0.605
Drinking, n (%) Y N	49 (65.33) 26 (34.77)	28 (48.28) 30 (51.72)	8 (47.06) 9 (52.94)	χ²=0.008	0.930
Location, n (%) Oral cavity Oropharynx Hypopharynx Larynx	26 (34.70) 24 (32.00) 18 (24.00) 7 (9.30)	21 (36.20) 17 (29.30) 14 (24.10) 6 (10.30)	5 (29.40) 7 (41.20) 4 (23.50) 1 (5.90)	χ²=1.040	0.792
Radiotherapy Y N	45 (60.00) 30 (40.00)	36 (62.07) 22 (37.93)	9 (52.94) 8 (47.06)	χ²=0.456	0.499
Surgery, (%) Y N	25 (33.33) 50 (66.67)	19 (32.76) 39 (67.24)	6 (35.29) 11 (64.71)	χ²=0.038	0.845
T Stage, n (%) T1 T2 T3 T4	2 (2.67) 6 (8.00) 26 (34.67) 45 (60.00)	2 (3.45) 6 (10.34) 16 (27.59) 34 (58.62)	0 (0.00) 0 (0.00) 6 (35.29) 11 (64.71)	/	/
N Stage, n (%) N0 N1 N2 N3	15 (20.00) 6 (8.00) 31 (41.33) 23 (30.67)	12 (20.69) 3 (5.17) 24 (41.38) 19 (32.76)	3 (17.65) 3 (17.65) 7 (41.18) 4 (23.53)	/	/

-:Fisher exact; χ²: Chi-square test.

### 4.2 Efficacy analysis

#### 4.2.1 Efficacy evaluation per RECIST 1.1 criteria

Imaging data of patients before and after neoadjuvant therapy were collected. Efficacy was evaluated for measurable lesions (primary tumor + radiologically positive regional lymph nodes) in the study group and measurable lesions in the control group respectively, in accordance with RECIST 1.1. Efficacy was classified into four grades (clinical CR, PR, SD, PD), and ORR and DCR were calculated.

Results showed that in the study group, there were 3 cases of CR (5.17%), 42 cases of PR (72.41%), 11 cases of SD (18.97%), and 2 cases of PD (3.45%), with ORR of 45 cases (77.59%) and DCR of 56 cases (96.55%). In the control group, there were 0 cases of CR, 9 cases of PR (52.94%), 6 cases of SD (35.29%), and 2 cases of PD (11.76%), with ORR of 9 cases (52.94%) and DCR of 15 cases (88.24%).

**Figure 2:**
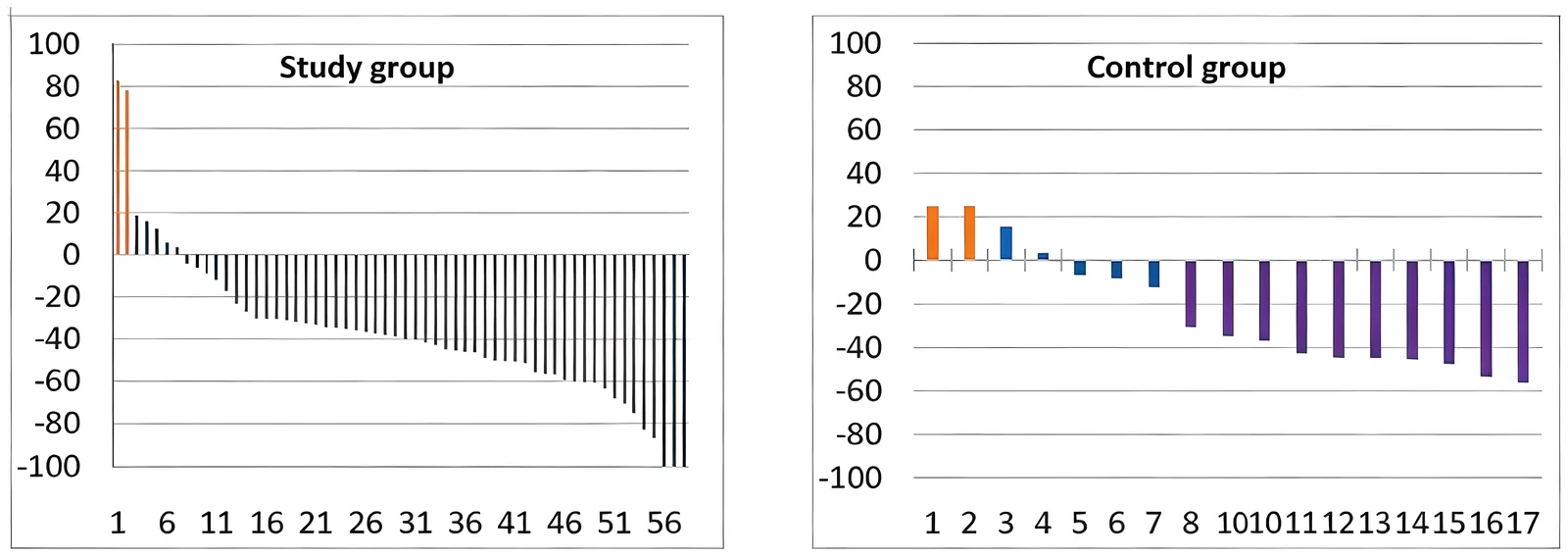
Waterfall plot of primary tumor regression rate in both groups based on RECIST1.1.

Statistical analysis revealed no statistically significant differences in CR, PR, SD, PD, ORR, or DCR between the two groups (P > 0.05). However, the study group showed a trend of superiority in ORR over the control group (77.59% vs 52.94%, χ²=2.833, P = 0.078), and the small sample size may have affected the stability of statistical results. A waterfall plot was drawn based on the percentage of target lesion regression before and after neoadjuvant therapy to intuitively demonstrate treatment efficacy (**Figure 2**). Efficacy of neoadjuvant therapy on primary tumors and positive regional lymph nodes in both groups evaluated by RECIST 1.1 is detailed in **Table 3**.

**Table 3:** RECIST 1.1 assessment of recent efficacy in study and control groups.

Recist1.1	Total (n=75)	Study group (n=58)	Control group (n=17)	χ²	P
CR, n (%)	3 (4.00)	3 (5.17)	0 (0.00)	–	1.000
PR, n (%)	51 (68.00)	42 (72.41)	9 (52.94)	0.382	0.536
SD, n (%)	17 (22.67)	11 (18.97)	6 (35.29)	1.177	0.278
PD, n (%)	4 (5.33)	2 (3.45)	2 (11.76)	–	0.219
ORR, n (%)	54 (72.00)	45 (77.59)	9 (52.94)	3.11	0.078
DCR, n (%)	71 (94.67)	56 (96.55)	15 (88.24)	–	0.219

-:Fisher exact;χ²: Chi-square test.

### 4.3 Pathological downstaging following neoadjuvant therapy and surgery

In this study, patients underwent efficacy evaluation and assessment for surgical indications after every two cycles of neoadjuvant therapy. A total of 25 patients who were deemed eligible for surgery and provided informed consent subsequently underwent surgical resection within 4 to 6 weeks after completing neoadjuvant therapy (see **Table 2**). These included 19 patients from the study group and 6 from the control group. All surgical procedures consisted of resection of the primary tumor combined with regional neck lymph node dissection.

Among the 19 surgical patients in the study group, efficacy evaluation according to RECIST 1.1 criteria indicated that 15 achieved a partial response (PR) and 4 had stable disease (SD). In the control group, among the 6 patients who underwent surgery, 3 were evaluated as PR and 3 as SD.

Analysis of pathological downstaging, based on the examination of resected specimens (primary tumor and lymph nodes), revealed reductions in both T and N stages in a proportion of patients across both groups. Specifically, in terms of T-stage downstaging, 9 patients (47.37%) in the study group achieved pCR in the primary tumor specimen. Regarding N-stage downstaging, 4 patients (21.05%) in the study group were down-staged from node-positive (N+) to node-negative (N0) status. In contrast, no patients in the control group achieved pCR in the target lesions, and only 1 patient (16.67%) exhibited any pathological downstaging. The overall pathological downstaging rate was significantly higher in the study group compared to the control group (57.89% vs. 16.67%, p=0.032). (For details, see **Tables 4**, **5**).

**Table 4:** Pathological changes of the study and control groups before and after operation.

Pathological downstaging assessment	n(%)	Study group n(%)	Control group n(%)	χ²	P
Surgery, n	25	19	6		
Downstaging, n (%)	12 (48.00)	11 (57.89)	1 (16.67)	4.59	0.032
Primary pCR, n (%)	10 (40.00)	9 (47.37)	0 (0.00)	/	/
Lymphnode pCR, n (%)	4 (16.00)	4 (21.05)	0 (0.00)	/	/

χ²: Chi-square test.

**Table 5:** Comparison of T and N stages in 12 patients with pathological decline.

cTNM	ypTNM	Study group (n=11)	Control group (n=1)
Primary downstaging			
cT stage	ypT stage		
T2	ypT1	1	0
T3	ypT0	3	0
	ypT1	1	0
T4	ypT0	6	0
	ypT2	0	1
Total	12	11	1
pCR patient(n)		9	0
Lymph downstaging			
N stage	ypN stage		
N1	N0	1	0
N2	N1 N0	0 2	0 0
N3	N2 N1 N0	2 1 1	0 0 0
Total	7	7	0
pCR patient (n)		4	0

### 4.4 Safety evaluation

Adverse reaction data during neoadjuvant therapy were obtained by reviewing patients’ inpatient medical records, outpatient medical records, and follow-up data. Safety was evaluated in accordance with the CTCAE Version 5.0. Results showed that among all enrolled patients, the incidence of grade 1–2 adverse reactions was 86.67%, mainly including leukopenia in 26 cases (34.67%), erythrocytopenia in 20 cases (26.67%), elevated transaminases in 6 cases (8.00%), alopecia in 6 cases (8.00%), gastrointestinal reactions (nausea, vomiting, diarrhea) in 5 cases (6.67%), rash in 2 cases (2.67%), and hypothyroidism and elevated bilirubin in 1 case each (1.33%).The incidence of grade 3–5 adverse reactions was 14.67%, including myelosuppression in 4 cases (5.33%), leukopenia in 4 cases (5.33%), erythrocytopenia in 2 cases (2.67%), elevated transaminases in 2 cases (2.67%), and pneumonia in 1 case (1.33%). Immunotherapy-related AEs in the study group mainly included hypothyroidism, rash, and elevated bilirubin, with 1 case each (1.72%), all of which were grade 1-2. There was no statistically significant difference in adverse reactions between the study group and the control group (P > 0.05). Detailed incidence of adverse reactions is shown in **Table 6**.

**Table 6:** Comparison of adverse events of neoadjuvant therapy between the study and control groups.

Adverse reactions	Total (n=75)	Study group (n=58)	Control group (n=17)	χ²	p
Grade 1-2, n (%)	65 (86.67)	49 (84.59)	16 (94.12)	0.76	0.384
Alopecia	6 (8.00)	5 (8.62)	1 (5.88)	0.00	1.000
Gastrointestinal reactions	5 (6.67)	3 (5.17)	2 (11.76)	0.16	0.658
Rash	2 (2.67)	1 (1.72)	1 (5.88)	0.01	0.936
Hypothyroidism	1 (1.33)	1 (1.72)	0 (0.00)	/	/
Myalgia	2 (2.67)	2 (3.45)	0 (0.00)	/	/
Leukopenia	26 (34.67)	19 (32.76)	7 (41.76)	0.41	0.521
Erythropenia	20 (26.67)	13 (22.41)	7 (41.76)	1.50	0.220
Thrombocytopenia	2 (2.67)	1 (1.72)	1 (5.88)	0.01	0.392
Elevated transaminase	6 (8.00)	5 (8.62)	1 (5.88)	0.00	1.000
Elevated bilirubin	1 (1.33)	1 (1.72)	0 (0.00)	/	/
Grade 3–5, n (%)	11 (14.67)	7 (12.07)	4 (23.53)	0.62	0.433
Leukopenia	4 (5.33)	2 (1.72)	2 (58.88)	–	0.219
Erythropenia	2 (2.67)	2 (1.72)	0 (0.00)	/	/
Myelosuppression	4 (5.33)	2 (1.00)	2 (11.76)	–	0.219
Pneumonia	1 (1.33)	1 (1.72)	0 (0.00)	/	/
Elevated transaminase	2 (2.67)	2 (1.72)	0 (0.00)	/	/

-:Fisher exact.

### 4.5 Survival analysis

Follow-up was conducted until March 2025. Eight patients were lost to follow-up in the study group, compared to one in the control group. Survival analysis indicated a mean follow-up time of 35.72 months (95% CI: 30.87–40.58) for the entire cohort. The median overall survival (OS) in the control group was 27 months (95% CI: 16.02–37.98), whereas the median OS had not been reached in the study group by the end of the follow-up period. A total of 9 deaths occurred in the study group and 10 in the control group, all attributable to disease progression.

**Figure 3:**
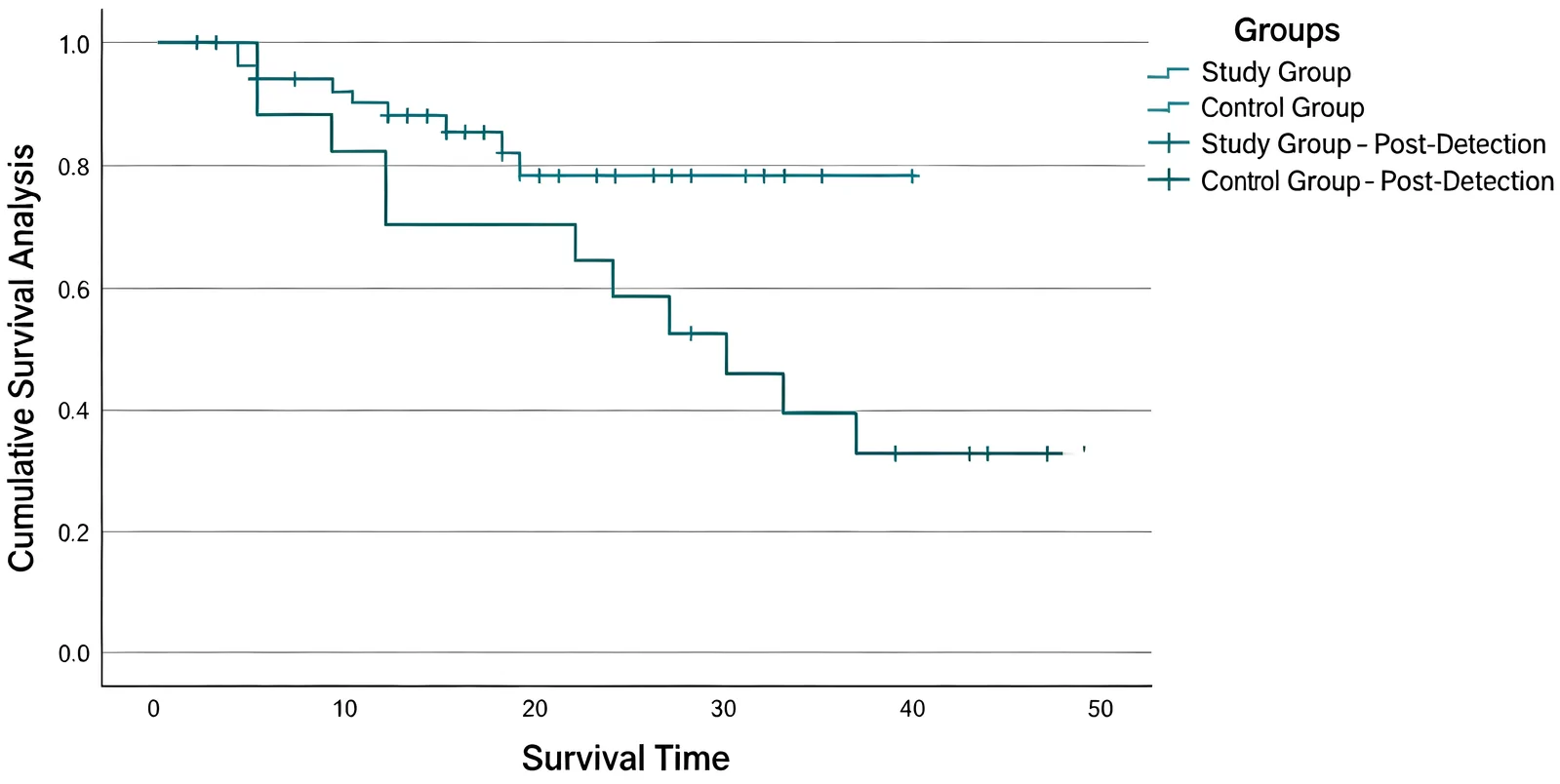
Kaplan-Meier curve of overall survival.

**Figure 4:**
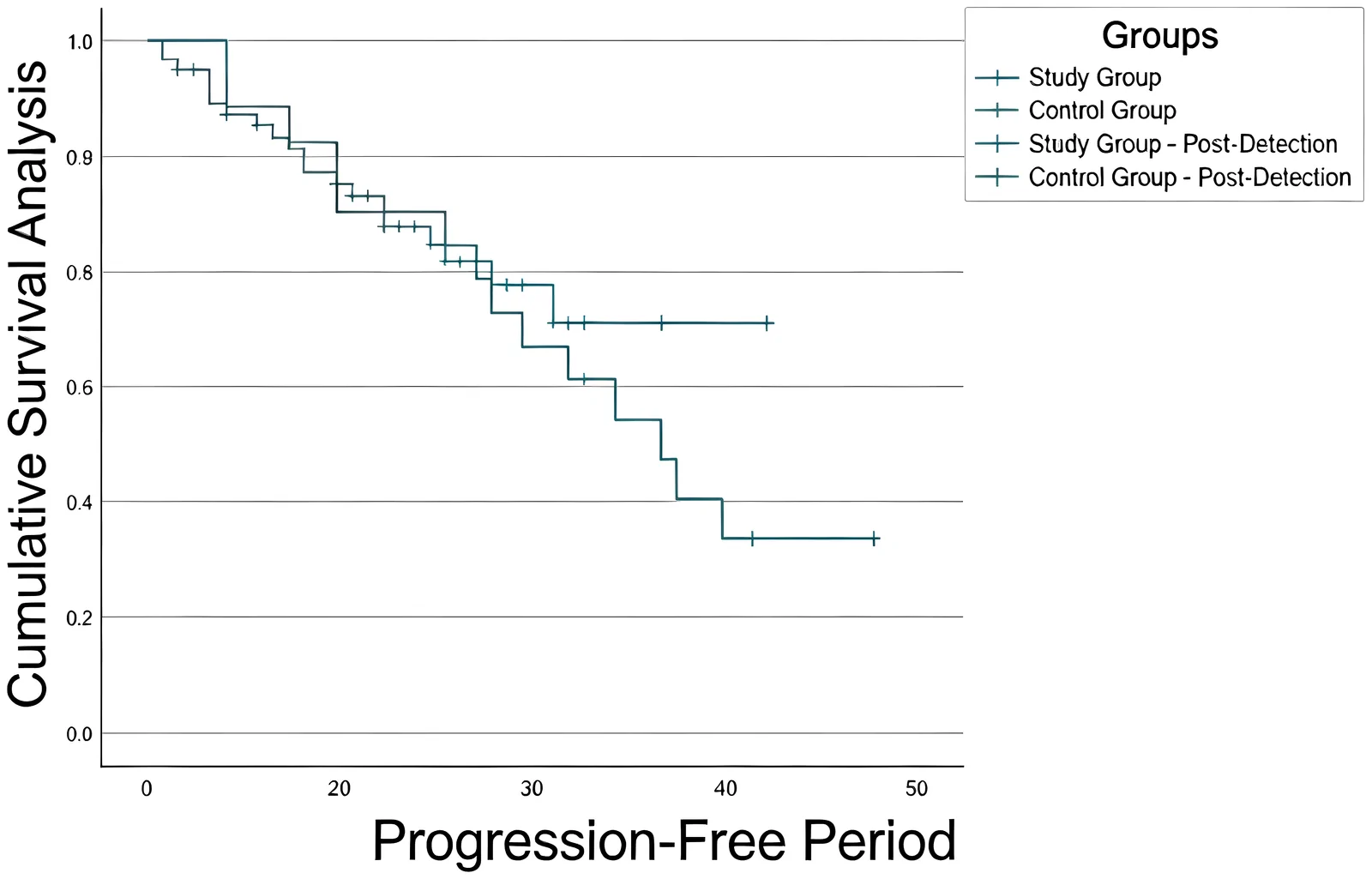
Kaplan-Meier curve of progression free survival.

As shown in **Figure 3**, the difference in the survival curves between the two groups was statistically significant (p = 0.044). The 1-year and 3-year OS rates were higher in the study group than in the control group (1-year OS: 87.9% vs. 68.8%; 3-year OS: 77.9% vs. 42.9%; all p-values < 0.05). The progression-free survival (PFS) curves are presented in **Figure 4**; however, the difference between the groups was not statistically significant (p = 0.406).

To explore LA-HNSCC survival predictors, univariate analysis was conducted using these variables: age (>60/≤60 years), gender, BMI (>18.5/≤18.5), neoadjuvant regimen (chemoimmunotherapy vs. alone), surgery, radiotherapy, smoking and alcohol history. Univariate results showed radiotherapy (p=0.039), neoadjuvant strategy (p=0.052) and surgery (p=0.006) were potentially prognostic factors.

Variables with a p-value < 0.1 in the univariate analysis were subsequently included in a multivariate Cox proportional hazards model. Multivariate analysis identified the neoadjuvant treatment strategy (p = 0.032) and surgery (p = 0.005) as factors associated with improved OS. These should be interpreted as exploratory given the sample size and group imbalance. Patients who underwent surgery had a lower risk of death (HR = 0.112, 95% CI: 0.024–0.522, p = 0.005). However, this result should be interpreted cautiously, as the surgical variable is subject to immortal time bias and the groups are imbalanced (58 vs. 17). Similarly, neoadjuvant chemoimmunotherapy was associated with a significantly lower risk of death compared to platinum-based chemotherapy alone (HR = 0.335, 95% CI: 0.123–0.910) (see **Table 7**). risk of death compared to platinum-based chemotherapy alone (HR = 0.335, 95% CI: 0.123–0.910) (see **Table 7**).

**Table 7:** Results of one-way analysis and multivariate analysis of COX model of prognosis of patients with LA HNSCC.

Variable	Univariate analysis						Multivariate cox regression analysis				
	B	SE	HR		95%CI	p	B	SE	HR	95%CI	p
Surgery	-2.078	0.754	0.125	0.029-0.549		0.006	-2.189	0.786	0.112	0.024-0.522	0.005
Neoadjuvant treatment regimen	-0.929	0.477	0.395	0.155-1.006		0.052	-1.095	0.510	0.335	0.123-0.910	0.032
Radiotherapy	-0.970	0.470	0.379	0.151-0.951		0.039	-0.389	0.515	0.678	0.247-1.861	0.450
Age	-0.801	0.497	0.449	0.170-1.187		0.107					
Sex	-0.934	1.034	0.393	0.052-2.982		0.366					
BMI	-0.449	0.476	0.638	0.251-1.623		0.346					
Smoking	0.231	0.466	1.260	0.505-3.141		0.620					
Drinking	0.067	0.460	1.070	0.434-2.636		0.884					

The surgery variable in the Cox model does not account for immortal time bias. Results should be interpreted with caution.

## 5 Discussion

HNSCC is one of the most common global malignant tumors with high malignancy and poor prognosis (23). Conventional treatments (surgery, radiotherapy, chemotherapy) for locally advanced HNSCC offer limited clinical benefits, with unsatisfactory efficacy, safety and long-term survival (24), highlighting the urgent need for novel therapeutic strategies. Neoadjuvant immunotherapy shows promising efficacy and safety across multiple cancers, significantly improving patient survival and prognosis (25). For platinum-refractory recurrent/metastatic HNSCC, PD-1 monotherapy also prolongs overall survival (26).

Tumor immunotherapy functions by blocking PD-1/PD-L1 binding: PD-1 inhibitors reverse T-cell exhaustion, restoring cytotoxic T lymphocyte-mediated tumor clearance (27). Chemotherapy and immunotherapy exert synergistic anti-tumor effects. Chemotherapy directly induces immunogenic cell death, boosts anti-tumor immunity and depletes immunosuppressive cells (28); immunotherapy amplifies immune responses to enhance chemosensitivity. Their combination remodels the tumor microenvironment, relieves immune tolerance, and sustains durable anti-tumor immunity (29, 30).

According to the data of this study, male patients accounted for 89.66%, with a male-to-female ratio of approximately 9:1, which was higher than the male-to-female risk ratio (3–4):1 reported in previous literature. This discrepancy in risk ratio may be attributed to the high prevalence of unhealthy habits such as tobacco and alcohol consumption among males in the Chaoshan region. The enrolled patients were predominantly middle-aged and elderly, and 51.50% of them were aged 60 years or older, which is consistent with the epidemiological characteristic that HNSCC occurs more frequently in elderly patients.

This study assessed neoadjuvant efficacy via tumor and positive lymph node diameter measurements on imaging, per RECIST 1.1. Neoadjuvant therapy aims to achieve pathological tumor downstaging, boost surgical resection rate, preserve organ function and quality of life, clear micro-metastases, and improve long-term patient survival.

Target lesion efficacy assessment showed no significant between-group differences in CR, PR, SD, PD, ORR and DCR (all p>0.05). However, the neoadjuvant immunochemotherapy group had a higher pathological downstaging rate (57.89% vs. 16.67%). The study group had 6 (31.58%) overall pCR cases and 9 (47.37%) primary tumor-only pCR cases, while the control group had only 1 downstaging case and zero pCR.

The underlying mechanism may involve immune cell infiltration in the tumor microenvironment after immunotherapy, mediating tumor killing without obvious shrinkage and even pseudoprogression. This explains non-significant differences between group in RECIST 1.1 imaging endpoints (CR, PR, SD, PD, ORR, DCR), despite a higher pathological downstaging rate in the study group. Postoperative analysis also showed better pathological response in primary tumors than cervical lymph nodes, likely due to distinct immune microenvironments.

A previous single-arm, phase II clinical trial in HNSCC evaluated the efficacy and safety of neoadjuvant chemotherapy combined with camrelizumab in patients with locally advanced HNSCC, all of whom underwent surgical resection 3 weeks later. The clinicopathological downstaging rate was 100% and the pCR rate was 37.0%, showing a consistent trend with the present study. Another study reported a pCR rate of 42% in locally advanced HNSCC patients receiving neoadjuvant therapy with nivolumab plus carboplatin and paclitaxel. However, direct horizontal comparison is infeasible due to differences in baseline characteristics of enrolled patients and study endpoints, which can only serve as references for clinical therapeutic regimens. Further large-scale studies are still required to confirm the exact efficacy of neoadjuvant immunochemotherapy in HNSCC.

This study showed a statistically significant difference in overall survival (OS) between the two groups (p = 0.044), indicating neoadjuvant immunochemotherapy improves prognosis in locally advanced HNSCC. Multivariate Cox regression identified neoadjuvant regimen (p = 0.032) and surgery (p = 0.005) as factors associated with improved OS. In multivariate analysis, surgery was associated with a lower risk of death (HR = 0.112, p = 0.005), as was neoadjuvant immunochemotherapy (HR = 0.335, p = 0.032). Radiotherapy was significant in univariate analysis but not an independent prognostic factor in multivariate analysis. Immuno-chemo therapy improves prognosis in locally advanced HNSCC. Multivariate Cox regression identified neoadjuvant regimen (p = 0.032) and surgery (p = 0.005) as factors associated with improved OS. Immunochemotherapy improves prognosis in locally advanced HNSCC. Multivariate Cox regression identified neoadjuvant regimen (p = 0.032) and surgery (p = 0.005) as factors associated with improved OS. However, several methodological limitations warrant caution in interpreting these results. In multivariate analysis, surgery was associated with a lower risk of death (HR = 0.112, p = 0.005), as was neoadjuvant immunochemotherapy (HR = 0.335, p = 0.032). These results are preliminary, as the analysis does not account for immortal time bias in the surgical variable, and the two groups are severely unbalanced (58 vs. 17). Radiotherapy was significant in univariate analysis but not an independent prognostic factor in multivariate analysis.

These findings suggest neoadjuvant immunochemotherapy may improve OS in LA-HNSCC, though confirmation in larger studies is needed. Whether surgery in appropriate candidates after neoadjuvant therapy further improves outcomes warrants further investigation. Patient preferences and quality of life should also be considered in treatment decisions.

On the basis of efficacy evaluation, this study also assessed the safety of different neoadjuvant therapeutic regimens. Previous literature reported that adverse events (AEs) of immune checkpoint inhibitors were predominantly grade 1–2, while grade 3–4 AEs were rare (31) The main AEs of PD-1/PD-L1 inhibitors include pneumonitis and hypothyroidism (32) Different types of immune checkpoint inhibitors induce distinct AEs: for instance, nivolumab mainly causes endocrine AEs (17), pembrolizumab frequently leads to arthritis, pneumonitis, and hepatic insufficiency, and capillary proliferation is the most common AE of camrelizumab (11).

In the present study, patients with LA-HNSCC received various PD-1 inhibitors. The main AEs of neoadjuvant therapy included leukopenia in 26 cases (34.67%), erythropenia in 20 cases (26.67%), elevated transaminase in 6 cases (8.00%), alopecia in 6 cases (8.00%), gastrointestinal reactions (including nausea, vomiting, and diarrhea) in 5 cases (6.67%), and rash in 2 cases (2.67%). There was no statistically significant difference in the incidence of AEs between the study group and the control group. Moreover, most AEs were grade 1–2, and the incidence of severe AEs was extremely low, which was consistent with previous research data, indicating that neoadjuvant immunochemotherapy is a safe and feasible therapeutic strategy.

## 6 Conclusion and limitations

This study has several limitations. First, the sample size is small (n = 75), and the two groups are severely unbalanced (58 vs. 17), which limits statistical power and may introduce residual confounding. Second, the surgical variable in the Cox model is subject to immortal time bias: patients must survive long enough to undergo surgery, which may inflate the apparent survival benefit. We chose not to perform *post hoc* sensitivity analyses (such as landmark analysis or time-dependent Cox) because, with only 17 control patients, any such analysis would be underpowered and could produce misleading estimates. Third, systematic HPV/p16 testing was performed only for oropharyngeal cases with non-keratinizing morphology, per AJCC 8th edition recommendations, and PD-L1 CPS testing was not routinely reimbursed for neoadjuvant indications during the study period. We were unable to retrieve systematic p16 or PD-L1 data from pathology records for inclusion in this analysis. The absence of these biomarkers limits our ability to adjust for known prognostic factors. Fourth, the PD-1 inhibitor regimens were heterogeneous (tislelizumab, pembrolizumab, and penpulimab), and the follow-up duration was unequal between groups. Despite these limitations, this study provides real-world data on neoadjuvant immunochemotherapy in LA-HNSCC and may inform the design of future prospective trials.

In this retrospective study of 75 patients with LA-HNSCC, neoadjuvant immunochemotherapy was associated with a higher pathological downstaging rate (57.89% vs. 16.67%, p = 0.032) and improved overall survival (p = 0.044) compared with chemotherapy alone, without increasing adverse events. However, our findings are preliminary and should be considered hypothesis-generating. Key limitations include a small sample size (n = 75), severe group imbalance (58 vs. 17), heterogeneous PD-1 inhibitor regimens, absence of systematic HPV/p16 and PD-L1 testing, immortal time bias in the surgical analysis, and unequal follow-up duration between groups. Larger prospective studies with systematic biomarker stratification are needed to confirm these results.

## Data Availability

The raw data supporting the conclusions of this article will be made available by the authors, without undue reservation.
